# Supplementary material for: Consensus and conflict among ecological forecasts of Zika virus outbreaks in the United States
Source: Sci Rep. 2018 Mar 21;8:4921. doi: 10.1038/s41598-018-22989-0 (PMC5862882; doi:10.1038/s41598-018-22989-0)
Supplement: Supplementary file 1 — Supporting Information [file 41598_2018_22989_MOESM1_ESM.pdf]

Supporting Information for: Consensus and conflict among ecological forecasts of Zika virus outbreaks in the United States

Colin J. Carlson[1,2*], Eric Dougherty[3], Mike Boots[4], Wayne Getz[3,5], and Sadie J. Ryan[6,7,8]

[1]National Socio-Environmental Synthesis Center, University of Maryland, Annapolis, MD 21401, USA.
[2]Department of Biology, Georgetown University, Washington, D.C. 20057, USA.
[3]Department of Environmental Science, Policy and Management, University of California Berkeley, Berkeley, CA 94720-3112, USA
[4]Department of Integrative Biology, University of California Berkeley, Berkeley, CA 94720-3112, USA
[5]Schools of Mathematical Sciences, University of KwaZulu, Natal, South Africa
[6]Schools of Life Sciences, University of KwaZulu, Natal, South Africa
[7]Department of Geography, University of Florida, Gainesville, FL 32601
[8]Emerging Pathogens Institute, University of Florida, Gainesville, FL, 32610
*Corresponding author: ccarlson@sesync.org

ABSTRACT

Below, we provide find supplemental information for the study.

Table 1. Epidemiological parameters for the SEIIAR & SEI models presented in the main text; values taken directly from from Table 1 of Gao *et al.*'s (2017) study.

Parameter	Description	Range
a	Mosquito biting rate ($mosquito^{-1}day^{-1}$)	$(0.3, 1)$
b	Mosquito to human transmission rate ($bite^{-1}$)	$(0.1, 0.75)$
c	Human to mosquito transmission rate ($bite^{-1}$)	$(0.3, 0.75)$
β	Human to human (sexual) transmission rate	$(0.001, 0.1)$
$1/\gamma_{h1}$	Infectious period (acute)	$(3, 7)$
$1/\gamma_{h2}$	Infectious period (convalescent)	$(14, 30)$
$1/\gamma_h$	Infectious period (asymptomatic)	$(5, 10)$
η	Exposed human to mosquito transmission proportion	$(5, 10)$
θ	Proportion symptomatic infections	$(0.1, 0.27)$
κ	Exposed human to human transmission proportion	$(0, 1)$
$1/\mu$	Mosquito lifespan (day)	$(4, 35)$
$1/\nu_h$	Intrinsic incubation period (day)	$(2, 7)$
$1/\nu_v$	Extrinsic incubation period (day)	$(8, 12)$
τ	Convalescent human to human transmission proportion	$(0, 1)$

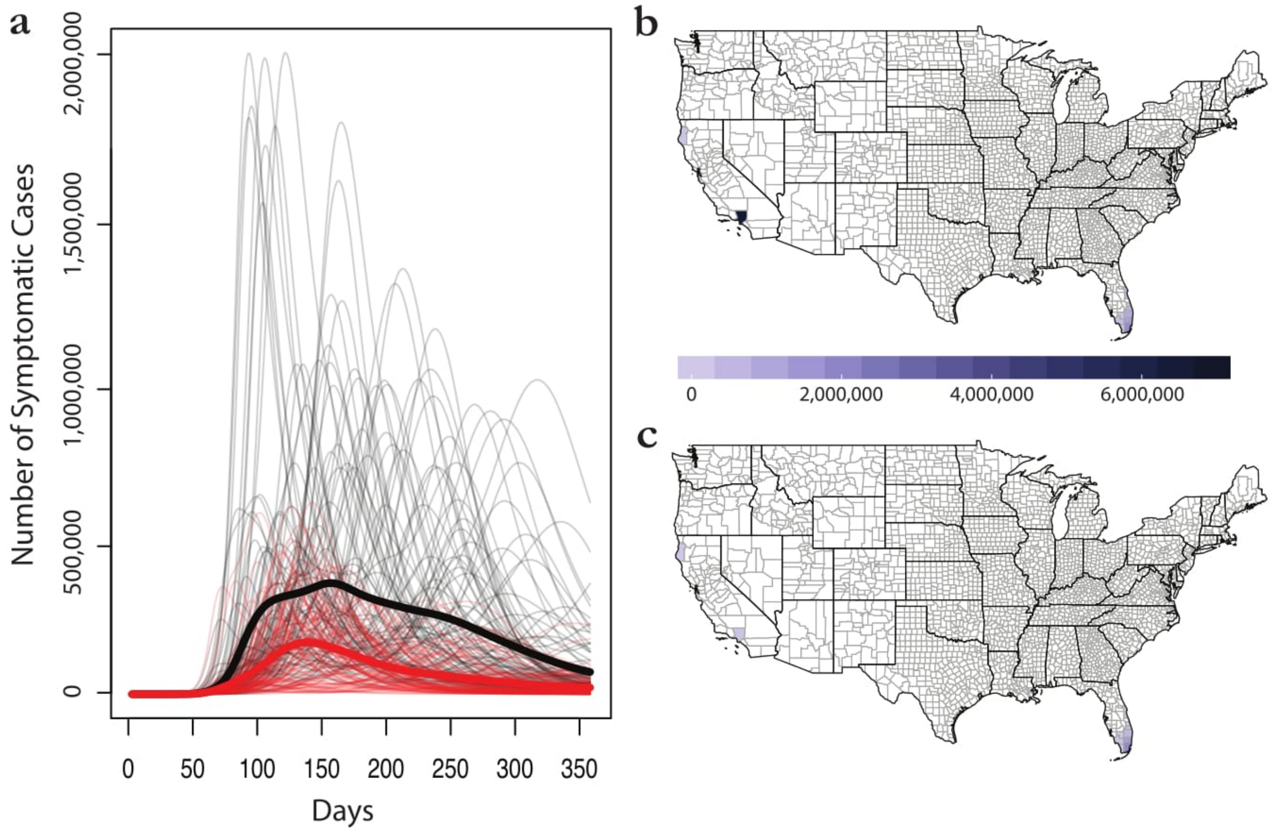

Figure 1. Full county versus partial population simulations with the Carlson model. Outbreak simulations (a) are given in black for the full county, and red for the partial county. Mean county case totals are mapped for the full county (b) and partial county (c). Maps were made in R 3.3.2[63] (https://www.R-project.org), using U.S. Census shapefiles.

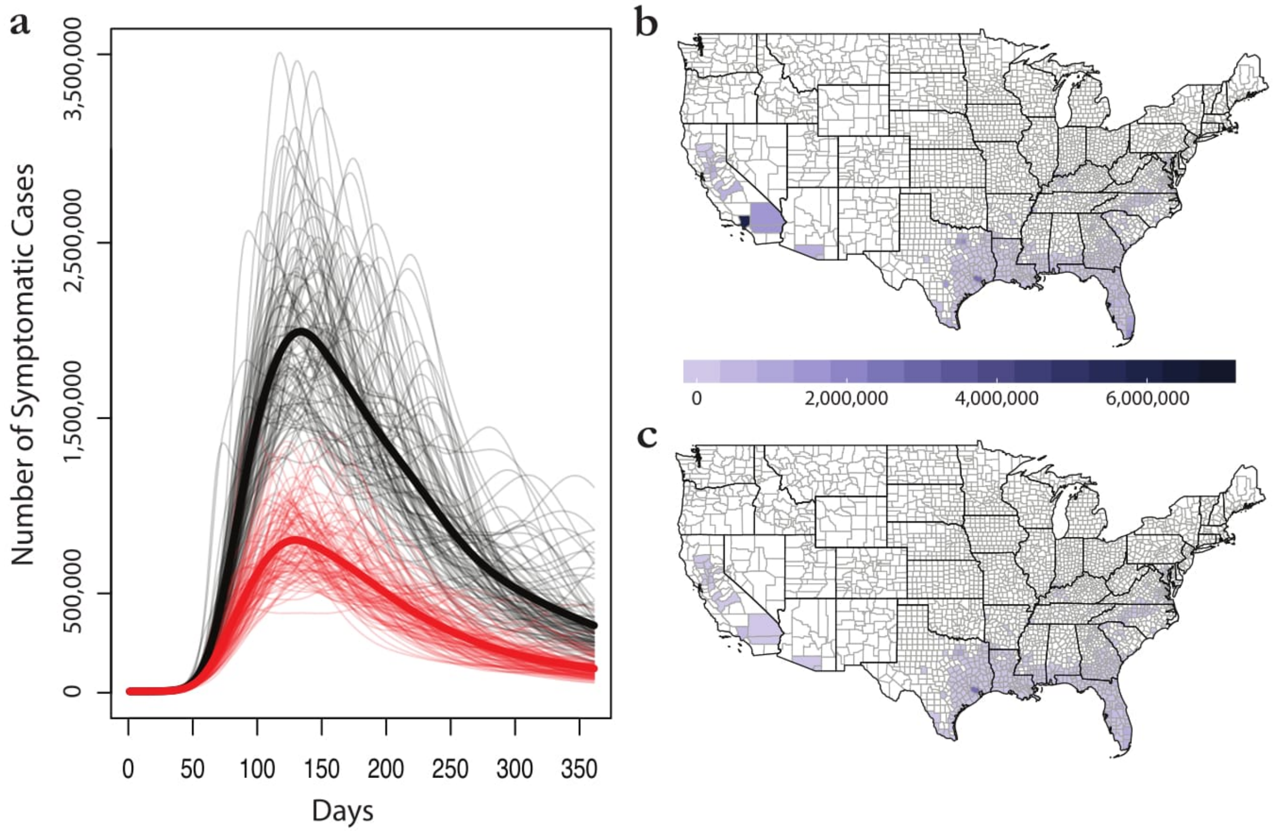

Figure 2. Full county versus partial population simulations with the Messina model. Outbreak simulations (a) are given in black for the full county, and red for the partial county. Mean county case totals are mapped for the full county (b) and partial county (c). Maps were made in R 3.3.2 [63] (https://www.R-project.org), using U.S. Census shapefiles.

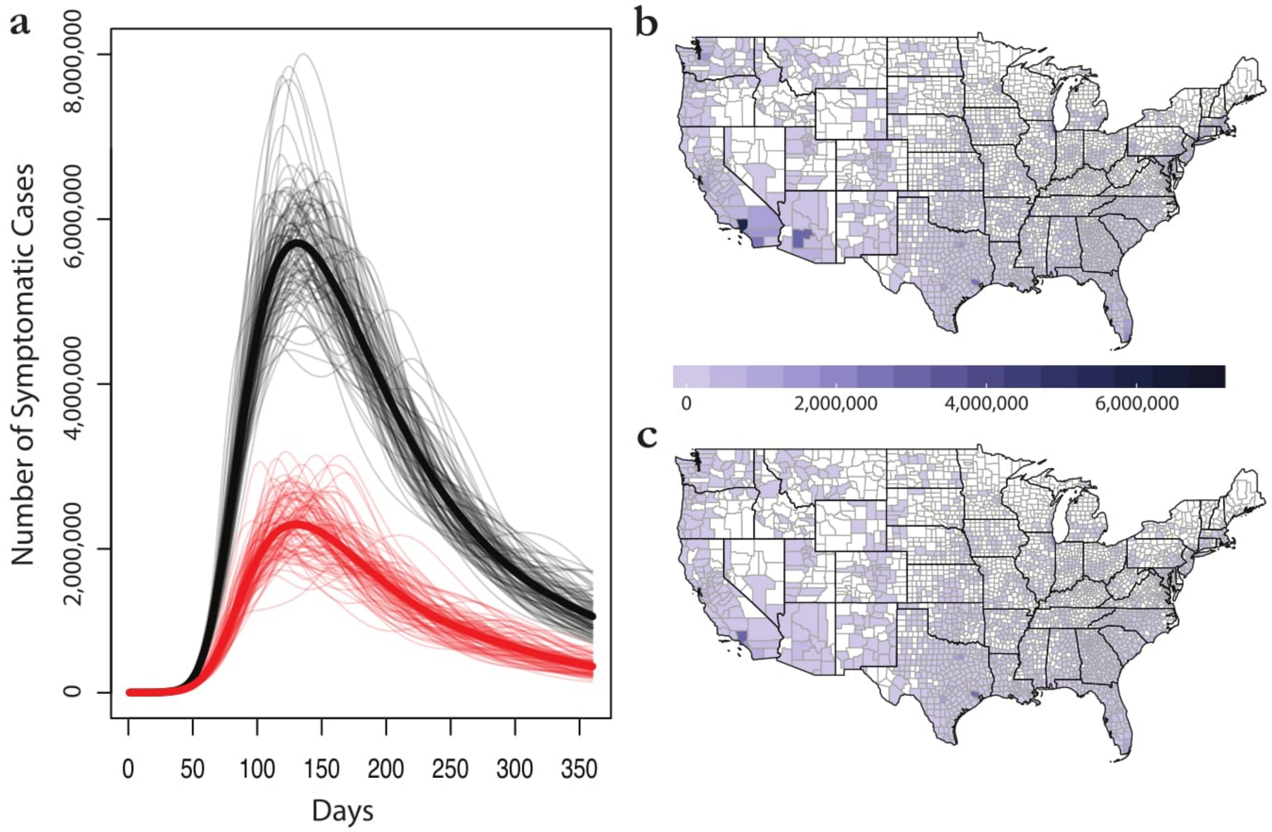

Figure 3. Full county versus partial population simulations with the Samy model. Outbreak simulations (a) are given in black for the full county, and red for the partial county. Mean county case totals are mapped for the full county (b) and partial county (c). Maps were made in R 3.3.2 [63] (https://www.R-project.org), using U.S. Census shapefiles.
